# The effects of exosomes derived from trabecular meshwork cells on Schlemm’s canal endothelial cells

**DOI:** 10.1038/s41598-021-01450-9

**Published:** 2021-11-09

**Authors:** Eri Takahashi, Junji Saruwatari, Tomokazu Fujimoto, Yuki Tanoue, Takaichi Fukuda, Toshihiro Inoue

**Affiliations:** 1 Department of Ophthalmology, Faculty of Life Sciences, Kumamoto University, 1-1-1 Honjo, Chuo-ku, Kumamoto, 860-8556 Japan; 2 Department of Pharmacology and Therapeutics, Graduate School of Pharmaceutical Sciences, Kumamoto University, 5-1 Oe Honmachi, Chuo-ku, Kumamoto, 862-0973 Japan; 3 International Research Center for Medical Sciences, Faculty of Life Sciences, Kumamoto University, 2-2-1 Honjo, Chuo-ku, Kumamoto, 860-0811 Japan; 4 Department of Anatomy and Neurobiology, Faculty of Life Sciences, Kumamoto University, 1-1-1 Honjo, Chuo-ku, Kumamoto, 860-8556 Japan

**Keywords:** Ocular hypertension, Extracellular signalling molecules

## Abstract

Trabecular meshwork (TM) and Schlemm’s canal (SC) are the main structures within the conventional outflow pathway, and TM cells and SC endothelial (SCE) cells are essential for controlling intraocular pressure. To examine the interaction between TM cells and SCE cells, we investigated whether exosomes contribute to intercellular communication. Additionally, TM cells in glaucoma acquire mesenchymal characteristics in response to transforming growth factor (TGF)-β2 and extracellular matrix proteins such as collagen type 1 (Col-1); these changes result in increased resistance of aqueous outflow. In this study, we stimulated TM cells with TGF-β2 and Col-1 and characterized the exosomal miRNAs (exomiRs) released in response to each stimulus. Isolated exosomes were rich in miRNAs, with downregulated miR-23a-5p and upregulated miR-3942-5p and miR-7515 levels following Col-1 or TGF-β2 stimulation. Next, a miRNA-mRNA network under TGF-β2 stimulation was constructed. There were no connections among the 3 miRNAs and predicted genes under Col-1 stimulation. GO and KEGG analyses revealed that the identified miRNAs were associated with various signaling pathways, including the inflammatory response. Interestingly, SCE cells treated with miR-7515 mimic showed increased *VEGFA*, *VEGFR2*, *PECAM*, and *Tie2* expression. Ultrastructures typical of exosomes and positive staining for exosomal markers were observed in human TM cells. Our data showed that TM cells may communicate with SCE cells via exomiRs and that miR-7515 may be important for SCE cell reprogramming.

## Introduction

Exosomes are endosome-derived extracellular vesicles (EVs) that contain many biomolecules, including lipids, proteins, and nucleic acids, such as microRNAs (miRNAs)^1^. The components in exosomes reflect the cell type and circumstances of the secreting cells, for example, cancerous, metastatic, ischemic, and so on^1–3^. When other cells take up exosomes, the bioactive components within these exosomes, especially exosomal miRNAs (exomiRs), play key roles in mediating several pathological changes, such as epithelial-mesenchymal transition, angiogenesis, and reprogramming, in the recipient cells^4^. In the eyes, exosomes released from retinal astrocytes and retinal pigment epithelial cells are associated with angiogenic phenomena^5,6^. In contrast, Dismuke et al. reported a method to isolate exosomes from the aqueous humor (AH); however, it is still unknown whether exosomes are associated with the pathogenesis of glaucoma^7^.

The conventional outflow of the AH is the pathway with flow via the trabecular meshwork (TM) and Schlemm’s canal (SC) into collector channels and the episcleral vein. TM cells control AH resistance by regulating the elasticity of the TM and phagocytosis^8^. In addition, in glaucoma, TM cells transform into myofibroblastic cells, which lose normal TM cell characteristics and overproduce extracellular matrix (ECM) components; as a result, the resistance of AH flow and thus intraocular pressure (IOP) increase^8^. Many reports show that transforming growth factor (TGF)-β2 is greatly involved in this phenomenon. The level of TGF-β2 is high in the AH of patients with glaucoma, and TGF-β2 increases the production of ECM components, such as fibronectin and collagen, and induces myofibroblastic changes in TM cells^9–12^. We previously reported that the integrin-associated ECM (fibronectin, collagen type 1 [Col-1], and laminin) is important for the acquisition of a mesenchymal phenotype in TM cells^13^. TM cells are located adjacent to SC endothelial (SCE) cells, and both are important in the regulation of AH outflow; however, how TM cells and SCE cells interact remains unclear. In this study, we hypothesized that the kind of exomiRs present depends on the status of TM cells, normal or glaucomatous/mesenchymal, and examined which miRNA is a key regulator of SCE cells.

## Results

### Normal and mesenchymal TM cells release exosomes

**Figure 1:**
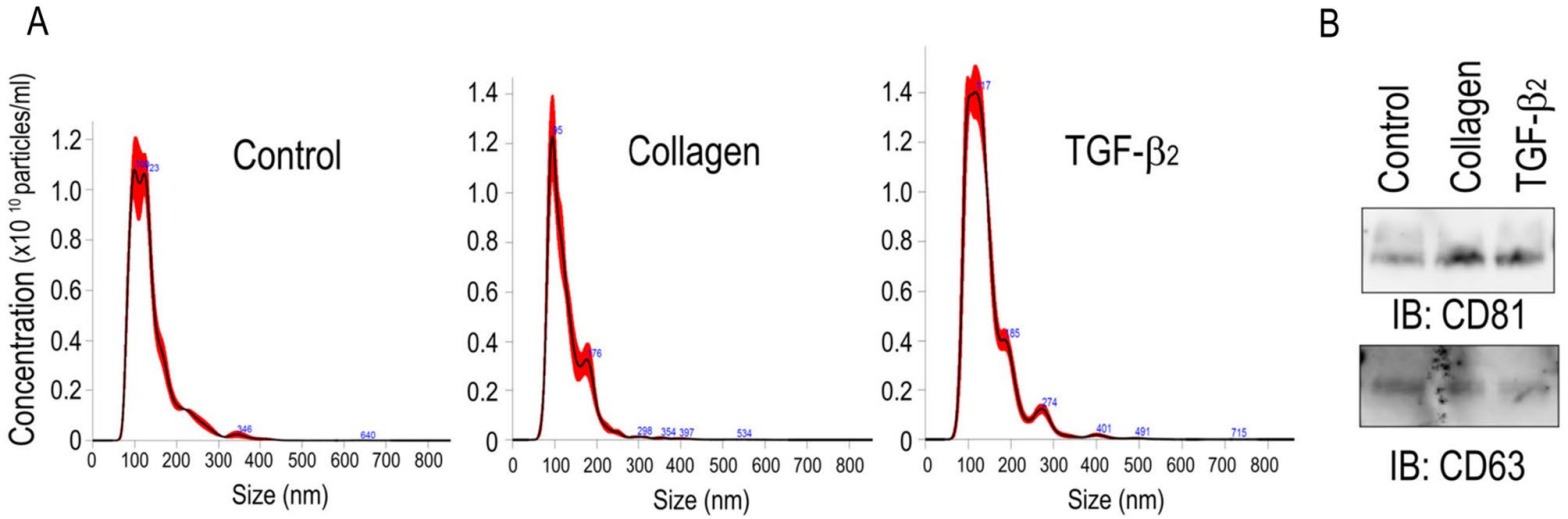
Purification of exosomes derived from monkey TM cells. Monkey TM cells were cultured in 10% exosome-free FBS with or without either Col-1 or TGF-β2 for 72 h, and exosomes in the supernatants were isolated using Exo-Quic-TC. (**A**) The concentration and size of exosomes were analyzed using a NanoSight analyzer. The standard deviation of five measurements of the same samples is shown in the red area. (**B**) Representative western blots of exosomes using antibodies against CD81 and CD63 (n = 3).

TM cells were isolated from the eyes of cynomolgus monkeys (*Macaca fascicularis*) and were stimulated with 5 ng/ml TGF-β2 or Col-1 to induce a mesenchymal phenotype^13^, and then exosomes were isolated from the cell supernatants using EXO-QUIC reagent. Nanoparticle tracking analysis using a NanoSight instrument was useful to visualize the nanosized particles and showed that the mean and mode size distributions of isolated particles ranged from 138.2 ± 1.5 and 104.1 ± 6.1 nm in the control group, 128.9 ± 5.1 and 96.0 ± 2.8 in the Col-1 group, and 144.2 ± 2.3 and 106.5 ± 5.2 in the TGF-β2 group, respectively (Fig. 1A). These particles expressed the specific exosomal markers CD63 and CD81 (Fig. 1B). Overall, TM cells released exosomes under control conditions and stimulation with TGF-β2 or Col-1.

### miRNA array analysis of exomiRs in exosomes derived from TM cells

**Figure 2:**
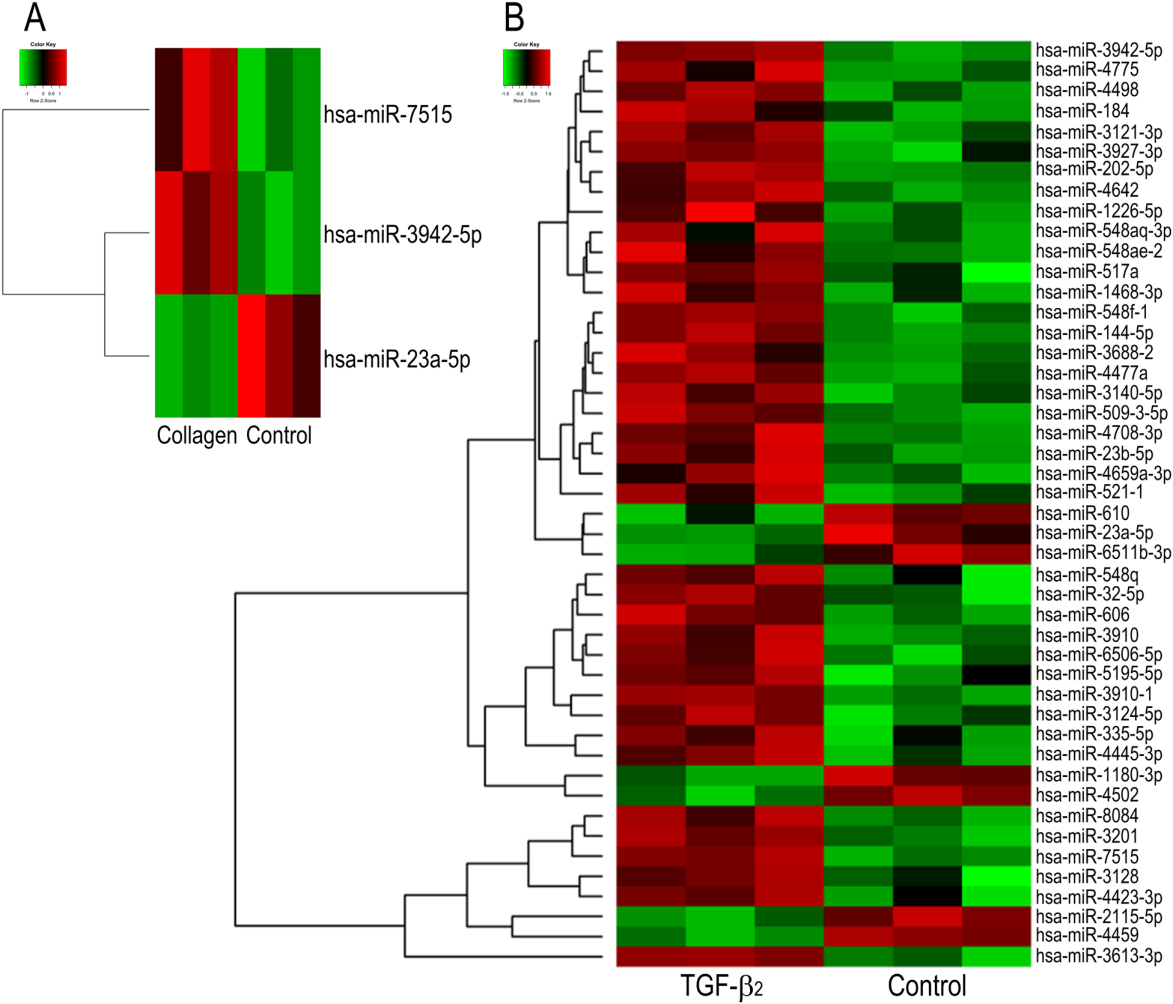
Heatmap of exosomal miRNA array analysis results for monkey TM cells. Cells were cultured in 10% exosome-free EBS with or without Col-1 (**A**) or TGF-β2 (**B**) for 48 h. The medium was changed, and the cells were cultured for an additional 48 h. Exosomes were collected from the latter 48-h supernatant using Exo-Quic-TC and subjected to an exosomal miRNA array (n = 3). Heatmaps were drawn using plots package of R version R version 4.0.5 (R Core Team (2021). R Foundation for Statistical Computing, Vienna, Austria.). The color key indicates that green represents low relative miRNA expression and red indicates high relative miRNA expression.

Figure 2 shows heatmaps of exomiRs in exosomes released from TM cells. Hsa-miR-3942-5p and hsa-miR-7515 levels were upregulated, while hsa-miR-23a-5p levels were downregulated in collagen vs. control exomiRs (Fig. 2A). In total, 39 miRNAs were upregulated, and 7 miRNAs were downregulated in exosomes from TGF-β2-treated TM cells compared to those from control cells (Fig. 2B). Three miRNAs altered in the collagen group were also found in the TGF-β2 group.

### Target gene networks and functional prediction of miRNAs in exosomes released from TM cells

**Figure 3:**
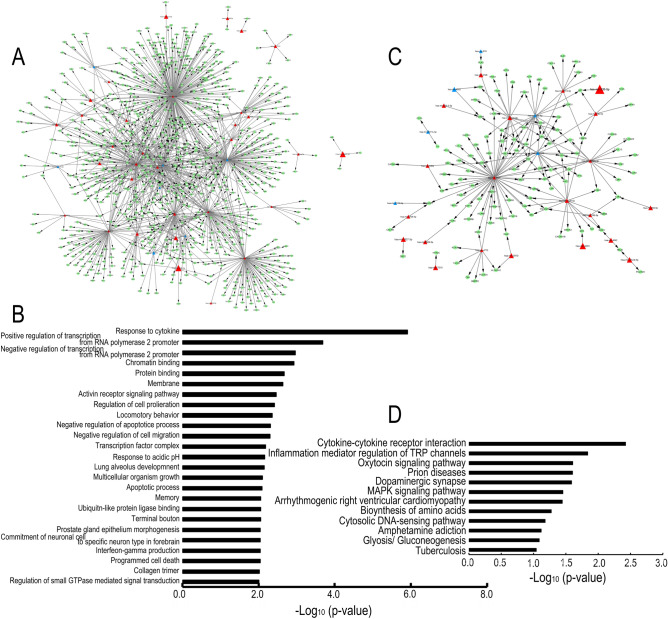
Exosomal miRNA bioinformatic analysis under TGF-β2 stimulation. (**A** and **C**) Network of miRNA-mRNA pairs associated with GO terms (**A**) and with KEGG pathways (**C**). Triangular and circular nodes represent miRNAs and predicted target mRNAs, respectively. High and low expression are represented in red and blue, respectively. (**B** and **D**) Histograms of significantly upregulated signaling pathways were generated according to the results of Gene Ontology (GO) (**B**) and Kyoto Encyclopedia of Genes and Genomes (KEGG) pathway analyses (**D**). The X-axis represents the logarithm of the p-value, and the Y-axis represents the name of the signaling pathways.

**Figure 4:**
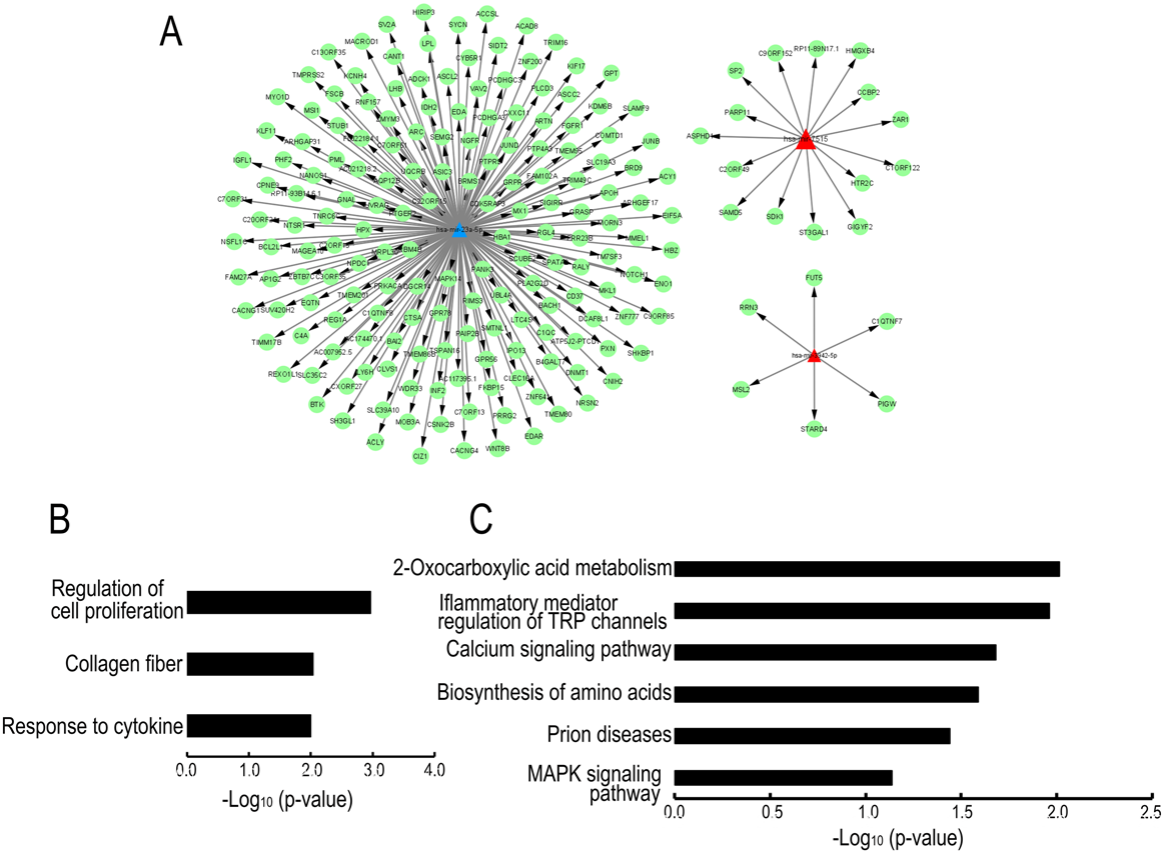
Exosomal miRNA bioinformatic analysis under Col-1 stimulation. (**A**) MiRNA-predicted target gene network. Triangular nodes represent upregulated miRNAs in red and downregulated miRNAs in blue. Green circles represent target genes. (**B** and **C**) Histograms of upregulated signaling pathways according to the results of GO (**B**) and KEGG pathway analyses (**C**). The X-axis represents the logarithm of the p-value, and the Y-axis represents the enriched pathway name.

A total of 1440 genes were predicted as target genes of 46 miRNAs, as shown in Fig. 2B. These miRNAs, except hsa-miR-7515 and hsa-miR-1468-3p, were connected in a miRNA-mRNA network constructed for TGF-β2-stimulated cultures (Supplementary Fig. 2). We performed GO and KEGG enrichment analyses to categorize the identified genes into biological functions. Figure 3A and C show a network of select miRNAs and mRNAs that were associated with the top GO and KEGG terms indicated in Fig. 3B and D, respectively (Fig. 3A and C). The target gene names are presented in Supplementary Table S1. We focused on three kinds of miRNAs that were commonly altered under TGF-β2 and Col-1 stimulation. MiR-7515 (increased) regulated some genes independently of other miRNAs; in contrast, miR-23a-5p (decreased) was centrally connected to several targets in both GO and KEGG pathways. MiR-3942-5p (increased) was associated only with C1q and tumor necrosis factor-related 7 (C1QTNF7) in GO analysis and did not regulate any of the genes in the KEGG pathways. The top 3 GO terms, “response to cytokine”, “negative regulation of transcription from RNA polymerase 2 promoter”, and “protein binding” (Fig. 3B), and the top 3 KEGG pathways, “cytokine-cytokine receptor interaction”, “inflammatory mediator regulation of transient receptor potential (TRP) channels”, and “oxytocin signaling pathway”, were found to be related to the target genes (Fig. 3D). On the other hand, in type 1 collagen-stimulated cultures, 193 genes were predicted as target genes of hsa-miR-7515, hsa-miR-3942-5p, and hsa-miR-23a-5p, and the miRNA-gene networks did not connect with each other (Fig. 4A). The GO analysis revealed that the predicted gene sets were involved in three pathways: “regulation of cell proliferation”, “collagen trimer”, and “response to cytokine” (Fig. 4B). According to the KEGG pathway analysis, the target genes were involved in “2-oxocarboxylic acid metabolism”, “inflammatory mediator regulation of TRP channels”, “calcium signaling pathway”, “biosynthesis of amino acids”, “prion diseases”, and “MAPK signaling pathway” (Fig. 4C).

### miR-7515 regulates lymphatic and angiogenic genes in SCE cells

**Figure 5:**
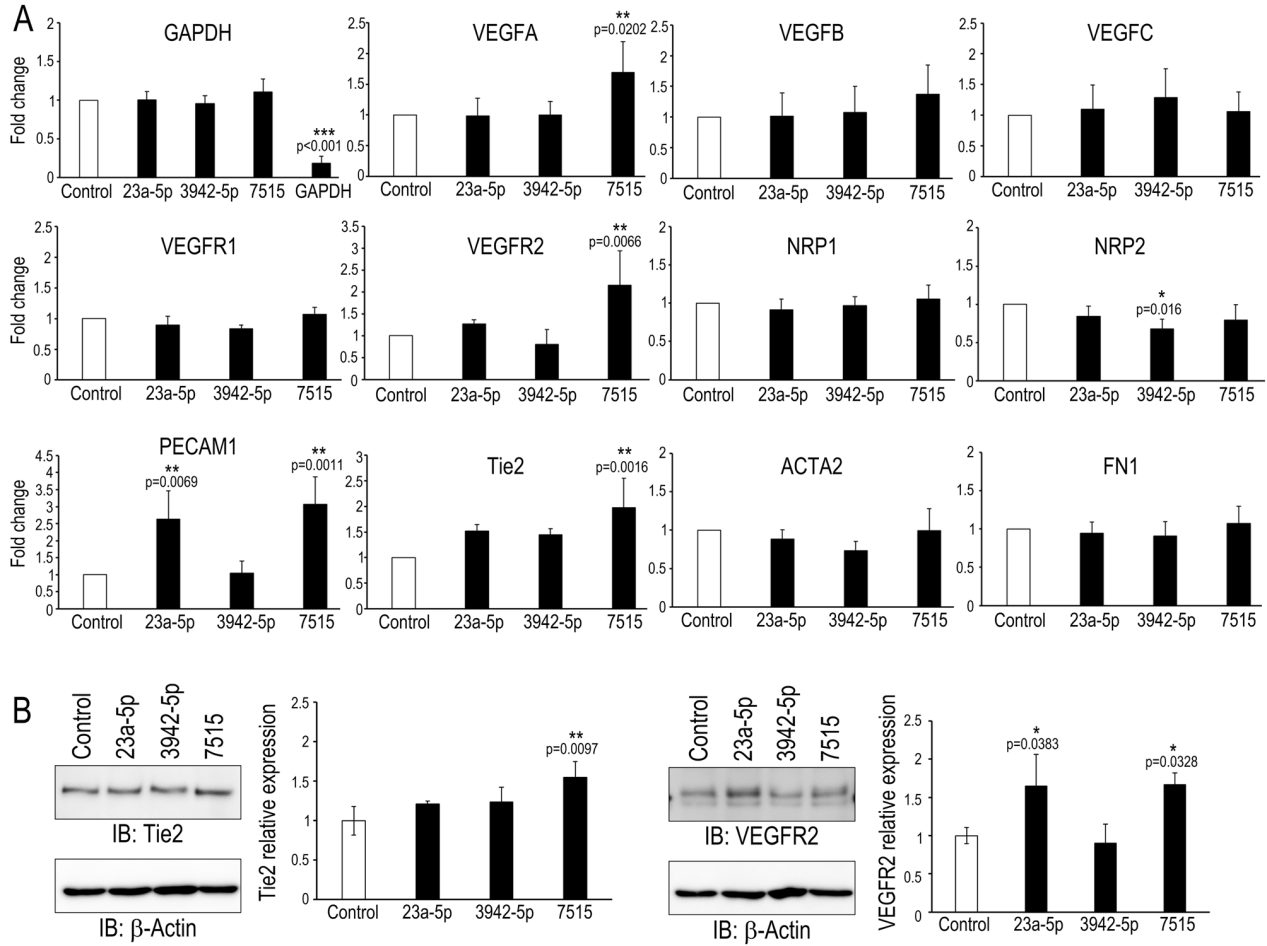
Effects of miRNA mimics on monkey SCE cells. (**A**) Quantitative RT-PCR was performed using the indicated primers to analyze monkey SCE cells transfected with miRNA mimics (miR-23a-5p mimic (23a-5p), miR-3942-5p mimic (3942-5p), miR-7515 mimic (7515)) or a miRNA positive control targeting GAPDH (control). Fold changes in the indicated mRNA expression relative to the level of the miRNA mimic control are presented (n = 4 for each group). *:*p* < 0.05, **:*p* < 0.01, ***:*p* < 0.001 by Dunnett’s test. VEGF: vascular endothelial growth factor, VEGFR: VEGF receptor, NRP: neuropilin, PECAM: platelet endothelial cell adhesion molecule, ACTA: α-smooth muscle actin, FN: fibronectin. (**B**) Western blot analysis of cell lysates after transfection with the indicated miRNA mimic. The graphs show the relative intensity (determined with Image J software) of the indicated proteins compared to β-actin, which was used as a loading control (n = 3). *:*p* < 0.05, **:*p* < 0.01 by Dunnett’s test.

Regarding the effects of the three identified miRNAs—miR-7515, miR-3942-5p, and miR-23a-5p—on SCE cells, we focused on endothelial-mesenchymal transition (Endo-MT) in SCE cells. We transfected miRNA mimics into SCE cells and performed qRT-PCR using primers for lymphatic, angiogenic, and Endo-MT markers (Fig. 5A). We found that the miR-7515 mimic promoted *VEGFA*, *VEGFR2*, *PECAM1*, and *Tie2* expression, the miR-23a-5p mimic increased *PECAM1* expression, and the miR-3942-5p mimic reduced *NRP2* expression. In addition, the levels of the mesenchymal markers *ACTA2* and *FN1* did not change in response to any miRNA mimic. Western blot analysis confirmed that Tie2 expression in SCE cells was increased by transfection with miR-7515 mimic and that VEGFR2 expression was increased by transfection with miR-23a-5p and miR-7515 mimics (Fig. 5B).

### Localization of exosomes in TM tissues from patients with glaucoma

**Figure 6:**
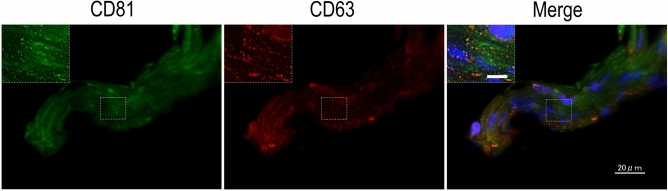
Exosome marker expression in human TM tissues. Representative images of exosome marker expression in excised TM tissues from patients with glaucoma (n = 4). CD81 is indicated in green, CD63 is indicated in red, and nuclei are indicated in blue (DAPI staining). Scale bars: 20 µm. The high-magnification image of the area enclosed by the white dotted square is shown in the upper left area of each panel. Scale bar: 5 µm.

**Figure 7:**
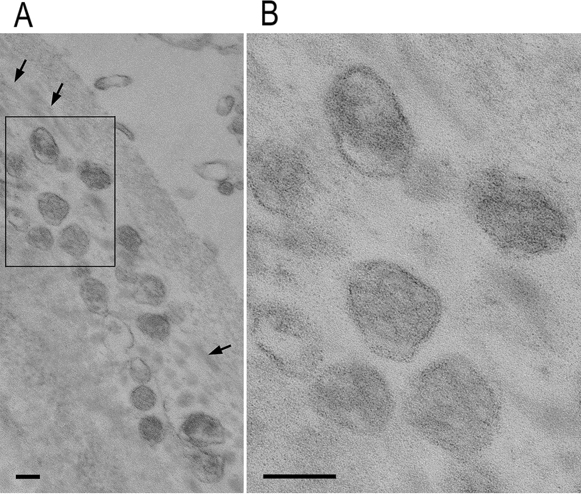
Transmission electron microscopy of human TM tissues. Transmission electron microscopy image of human TM tissue prepared from surgically transected material. (**A**) Exosomes are located in a cluster in the extracellular space. Arrows indicate collagen fibers. (**B**) Enlargement of the framed area in (**A**). Vesicles are enclosed by a limiting membrane that shows a typical profile of the lipid bilayer. Scale bars: 100 nm.

Finally, we turned our attention to exosome localization in human TM tissues. Immunohistochemistry analysis showed positive punctate staining for CD81 and CD63 localized around the nucleus, in the cytoplasm, and between the TM beams (Fig. 6). By using transmission electron microscopy, we confirmed the existence of characteristic vesicular structures in the extracellular space (Fig. 7A and B). They were enclosed by a limiting membrane, were 100–120 nm in diameter, and often clustered between the collagen fibers. These ultrastructural findings, combined with immunohistochemical localization of exosome markers, clearly indicated that exosomes were localized in human TM.

## Discussion

This study shows that TM cells secrete exosomes containing various miRNAs in response to TGF-β2 and Col-1 and that exomiRs are associated with the expression of lymphatic and angiogenic genes in SCE cells.

Inhibition of the TM-SC pathway cause an increase in IOP. The pathological changes in the TM in primary open-angle glaucoma (POAG) are the agglutination and narrowing of spaces between the TM beams and the deposition of ECM components^14^. During the development of these changes, TM cells undergo differentiation into myofibroblasts induced by TGF-β2 or connective tissue growth factor in the AH and alterations in the ECM components composing the basement membrane and TM beams in TM tissues^14,15^. Exosomes are secreted from various cell types, and TM cells release exosomes regardless of their static or mesenchymal cell phenotype (Fig. 1)^16^. Additionally, Dismuke et al. reported that glucocorticoids alter exosomal adhesiveness to the ECM of TM cells^17^, and we also revealed that exosomes from control and mesenchymal TM cells contained different miRNAs and that the variation in miRNAs under TGF-β2 stimulation was greater than that under Col-1 stimulation (Fig. 2). These data suggest that glaucomatous changes in the intraocular environment affect the kinds of components in exosomes. Interestingly, changes including downregulation of miR-23a-5p and upregulation of miR-3942-5p and miR-7515 by Col-1 stimulation were also identified following TGF-β2 stimulation (Fig. 2). In TM cells, the induction of the mesenchymal phenotype by Col-1 is associated with the TGF-β2 signaling pathway; on the other hand, TGF-β2 promotes the expression of Col-1^13,18^. Thus, TGF-β signaling and collagen-induced phenomena are interrelated, and the changes in miRNAs induced by Col-1 may be mediated by the TGF-β-Smad2/3 signaling pathway.

Not only TGF-β but also interleukin (IL)-8, IL-6, and tumor necrosis factor-α are increased in the AH in POAG, and Kaeslin et al. found that several inflammatory processes are involved in the upregulated signaling pathways in POAG by GO analysis based on a proteomic analysis of the AH^19–21^. Our GO and KEGG pathway analyses also revealed that inflammatory response terms, including “response to cytokines” and “cytokine-cytokine receptor interaction”, were at the top of the miRNA-mRNA network under TGF-β stimulation and that the terms “response to cytokine” and “inflammatory mediator regulation of TRP channels” were involved in Col-1 stimulation (Figs. 3 and 4). Thus, in glaucoma, TM cells are constantly exposed to environmental stimuli, leading to the epithelial-like-mesenchymal transition in TM cells, and mesenchymal/glaucomatous TM cells release exosomes containing miRNAs that transmit inflammatory factors and induce inflammatory responses to recipient cells.

There are no reports about the functions of these miRNAs in eyes, and there are only a few reports about the relationships between these miRNAs and systemic diseases. Downregulation of miR-23a-5p is suggested to regulate protective autophagy in leukemia, and miR-3942-5p is reported to be upregulated in systemic lupus erythematosus (SLE) patients. In addition, Lee et al. reported that miR-7515 is associated with lung cancer growth through the c-met signaling pathway^22–24^. SCE cells are a unique cell type and are thought to be similar to lymphatic endothelial cells. VEGFR2 and Tie2 are expressed during the development of SC, and Tie2 plays a key role in the integrity of adult SCE cells^25,26^. In addition, Endo-MT occurs in SCE cells, which is suggested to be one of the mechanisms of IOP elevation^27^. Therefore, we examined the relationship between these miRNAs and the pathology of glaucoma, focusing on Endo-MT and the change in permeability in SCE cells. We performed a transendothelial electrical resistance (TEER) assay to assess cell–cell contacts, and the assay showed that none of the three miRNA mimics (hsa-miR-23a-5p, hsa-miR-3942-5p, or hsa-miR-7515) changed the TEER in SCE cells (data not shown). In contrast, the hsa-miR-7515 mimic upregulated VEGFA, VEGFR2, PECAM1, and Tie2 mRNA levels and increased VEGFR2 and Tie2 expression (Fig. 5A and B). MiR-7515 was associated with top-ranking GO and KEGG pathways (Fig. 3A and C). Notably, miR-7515 was not incorporated in the miRNA-gene network under either TGF-β2 stimulation or Col-1 stimulation, even though the miRNA-mRNA network was extensive (because a miRNA can have thousands of target genes, and the targets often overlap due to pairing with sequences of only seven to eight bases) (Figs. 3 and 4)^28^. These data imply that TM cells sensing the glaucomatous anterior chamber environment induce the remodeling of SC to promote AH outflow via exomiR-7515. In addition, one of the predicted mRNAs associated with miR-7515 was CCBP2 (Figs. 3 and 4), which is expressed in lymphatic endothelial cells, and a single-nucleotide polymorphism in this molecule is associated with metastasis of breast cancer^29^, so any of the 15 mRNAs targeted by miR-7515 may be a candidate key regulator of AH flow. Furthermore, exosome uptake mechanisms include cell adhesion, fusion, micropinocytosis, phagocytosis, and receptor-mediated internalization^30^. Although we did not show a difference in the SCE cell uptake of exosomes released by static TM cells and those released by mesenchymal TM cells, exosomes may be an effective drug delivery system for targeting SCE cells if the mechanisms of preferential uptake into SCE cells are identified.

Analysis of TM tissue by transmission electron microscopy confirmed the existence of EV structures in surgically resected specimens (Fig. 7). Similarly, immunostaining analysis also showed exosome marker-positive punctate staining in the intercellular spaces, suggesting that exosomes localize in the TM (Fig. 6). Unfortunately, the endosomes and vesicles in the intercellular bilayer structure could not be identified because the intercellular structure was damaged by transmission electron microscopy analysis, probably due to the excision and removal process during surgery. Therefore, it was not possible to distinguish whether the EVs between the TM beams were derived from TM cells or in the AH in this study.

A limitation of this study was that we used cell lines isolated from cynomolgus monkeys (*Macaca fascicularis*). The number of miRNAs identified in *M. fascicularis* is no more than 1,000, and there are no available miRNA array probe sets for *M. fascicularis*. The sequences of miRNAs in *Macaca mulatta*, which are more available than those of miRNAs in *M. fascicularis*, are highly conserved in *M. fascicularis*, and Veeranagouda et al. reported that miRNAs in *M. fascicularis* are conserved in humans^31,32^. However, further experiments are needed to confirm whether the human analogs function the same in human eyes.

Our findings imply that exosomes released from mesenchymal TM cells are associated with maintaining SC homeostasis via exomiR-7515. Since TM cells may control AH outflow resistance by secreting exosomes, exosomes and exomiRs may be new targets for IOP regulation.

## Methods

### Cell culture and treatment

Enucleated eyes of cynomolgus monkeys were purchased (Shin Nippon Biomedical Laboratories, Kagoshima, Japan), and primary monkey TM cells and SCE cells were isolated as previously described^13,33^. In brief, SC was identified via insertion of a 6–0 nylon suture. TM tissues were gently extracted from the monkey eyes with fine forceps and cultured on collagen gel. After removing the TM tissues, pieces of the inner wall of SC were placed on collagen gel. TM and SCE cells were cultured in low-glucose DMEM (FUJIFILM Wako Pure Chemical, Osaka, Japan) supplemented with 10% fetal bovine serum (FBS), glutamine (2 mM), penicillin (100 U/mL), streptomycin (100 μg/mL), and amphotericin B (0.5 μg/mL) at 37 °C in 5% CO2. Cells passaged up to passage number 5 were used. Before experiments, cells were cultured with 10% exosome-depleted FBS (System Biosciences, CA, USA) for at least 24 h. The identity of TM cells was confirmed by identifying dexamethasone-induced myocilin expression using real-time polymerase chain reaction (RT-PCR).

### Antibodies and chemicals

Recombinant human TGF-β2 was purchased from R&D Systems (Minneapolis, MN, USA), and Col-1 was obtained from TOYOBO (Osaka, Japan). Antibodies against CD81 and β-actin were purchased from Santa Cruz Biotechnology (Dallas, TX, USA) and an anti-CD63 antibody was obtained from Abcam (Cambridge, UK). Antibodies against Tie2 and VEGFR2 were purchased from Cell Signaling Technology (Danvers, MA, USA).

### Isolation of exosomes

Cells were cultured on collagen-coated dishes^13^ or in the presence of 5 μg/ml TGF-β2 in medium supplemented with 10% exosome-depleted FBS for 72 h for nanoparticle tracking analysis (NanoSight, Quantum Design, Tokyo, Japan) and western blot analysis. For miRNA analysis, cells were pretreated for 48 h, the medium was changed, and then the cells were incubated for an additional 48 h. Supernatants were centrifuged at 3000×g for 15 min and filtered using a membrane filter with a pore size of 0.22 μm (Millipore, Burlington, MA, USA). The supernatants were mixed with Exo Quick-TC (System Biosciences, Mountain View, CA, USA) and incubated overnight at 4 °C according to the manufacturer’s protocol. Pellets containing exosomes were collected by centrifugation twice at 1500×g for 30 min. The exosome-containing pellets were subjected to analysis on a NanoSight LM10V-HS (outsourced to Quantum Design Japan, Tokyo, Japan), western blotting, and miRNA array analysis.

### Western blot analysis

Isolated exosome-containing pellets and whole SCE cells transfected with miRNA mimics were lysed in RIPA buffer and boiled for 10 min at 70 °C. Protein concentrations were assessed using the BCA Protein Assay Kit (Thermo Fisher Pierce, MA, USA), and equal amounts of proteins were loaded onto SDS-PAGE gels. After transfer to polyvinylidene difluoride membranes (Bio-Rad, Hercules, CA, USA), the membranes were incubated with primary antibodies, anti-CD63 and anti-CD81, at 4 °C overnight. The membranes were washed and further incubated with horseradish peroxidase-conjugated secondary antibodies for 60 min at room temperature. The western blot bands were visualized using chemiluminescence (Amersham Biosciences, Tokyo, Japan).

### MiRNA array analysis

RNA synthesis and whole-genome microarray analysis were outsourced to Filgen (Nagoya, Japan). Microarray analysis of miRNAs was performed using the Affymetrix GeneChip miRNA 4.0 Array (Thermo Fisher Scientific) in conjugation with the FlashTag Biotin HSR RNA Labeling Kit (Thermo Fisher Scientific). All reactions and hybridizations were carried out according to the manufacturer’s protocol. These arrays were washed with the GeneChip Fluidics Station 450 (Thermo Fisher Scientific) and scanned with the GeneChip Scanner 3000 (Thermo Fisher Scientific). Differentially expressed miRNAs were analyzed using the Transcriptome Analysis Console 4.0.2 (Thermo Fisher Scientific). To identify differentially expressed genes (DEGs), one-way analysis of variance was conducted, and gene lists were generated by applying a threshold fold change cutoff value of >|2| with a Benjamini–Hochberg false discovery rate (FDR) of < 0.05. We present the differentially expressed transcripts (DETs) in a heatmap with a dendrogram, where the distance metric is Euclidean; the distances between clusters were computed using the complete linkage method to profile gene expression patterns between the tested groups.

### Target prediction and functional annotation of miRNAs

Target prediction and functional annotation of miRNAs were performed using a previously reported procedure^34^. Target genes for differentially expressed miRNAs were identified using miRGate (http://mirgate.bioinfo.cnio.es/miRGate/), which processes data using 5 computational tools (miRanda, RNAhybrid, Pita, MicroTar and TargetScan)^35^. The total target genes of each miRNA were filtered by the intersection of at least 3 algorithms, and duplicate genes were removed for further analysis to prevent redundancy. Cytoscape software (http://www.cytoscape.org/index.html) was used to visualize the target genes of each miRNA. Next, we performed Gene Ontology (GO) enrichment analysis using DAVID (https://david.ncifcrf.gov/home.jsp, version 6.8) to categorize the predicted target genes into biological process terms^36^. We also performed pathway analysis using the Kyoto Encyclopedia of Genes and Genomes (KEGG) Pathway Database (http://www.genome.jp/kegg/) and DAVID to determine the main biological pathways in which the target genes are involved^37–39^. The p-value was the log-transformed value, and the fold enrichment was also calculated as the ratio of the number of genes in the gene set to the expected number in the category based on the human database.

### MiRNA mimic transfection and quantitative RT-PCR (qRT-PCR)

AccuTarget miRNA mimics for hsa-miRNA-23a, hsa-miRNA-3942, hsa-miRNA-7515, a positive housekeeping control, and a negative control were purchased from Bioneer (Daejeon, Korea). MiRNA mimics were transfected into SCE cells using Lipofectamine RNAiMAX (Thermo Fisher Scientific) according to the manufacturer’s protocol, and the culture medium was changed 6 h after transfection. After 24 h of treatment with 50 nM miRNA mimic, RNA was purified from SCE cells using NucleoSpin RNA (Macherey–Nagel, Düren, Germany) according to the manufacturer’s protocol. RNA was reverse transcribed using a PrimeScript RT-PCR kit (Takara Bio, Shiga, Japan) and subjected to qRT-PCR (TB Green Premix Ex Taq II; Takara Bio) using a StepOnePlus Real-time PCR system (Thermo Fischer Scientific). Fold changes in the expression of all target genes were calculated using the comparative CT method (ΔΔCT method), and β-actin was used as an endogenous control. The primer sequences are shown in Table 1. We conducted four independent experiments on all target genes. The results are expressed as the mean ± standard error. All data were analyzed using JMP statistical software (version 14.3.0; SAS Institute, Cary, NC, USA). Multiple groups were compared statistically using Dunnett’s multiple comparison test. In all analyses, differences were considered statistically significant at *p* < 0.05.

**Table 1:** Primer list for quantitative real-time polymerase chain reaction.

Primer name		Primer sequence (5′ to 3′)
VEGF-A	Forward	TCC ACC ATG CCA AGT GGT CC
	Reverse	CCA CCA GGG TCT CGA TTG GA
VEGF-B	Forward	GAA GCC GGA CAG CCC CAG
	Reverse	CTT GGC AAC GGA GGA AGC TG
VEGF-C	Forward	AGC AAC ACT ACC ACA GTG CCA
	Reverse	AGT CAT CTC CAG CAT CCG AGG
VEGFR1	Forward	GCA AAG CCA CCA ACC AGA AG
	Reverse	AGC CAC ACA GGT GCA TGT TA
VEGFR2	Forward	CCC AGA TGA CAA CCA GAC GG
	Reverse	TTG CTG GAC ACC ATT CCA CT
NRP1	Forward	TCT GCC ACT GGG AAC ATG AC
	Reverse	TGC CAT CTC CTG TGT GAT CC
NRP2	Forward	GGA TCA TCC TGC CCA GCT AC
	Reverse	AGC TGA GAT GGG TTC CAT GC
PECAM1	Forward	ACA CGG AAG TGC AAG TGT CCT
	Reverse	AGG GAG CCT TCC GTC CTA GAG
Tie2	Forward	ATA GGG TCA AGC AAC CCA GCC
	Reverse	GGT CCG CTG GTG CTT CAG AT
FN1	Forward	ACA AGC GTG TCT CTC TGC C
	Reverse	CCA GGG TGA TGC TTG GAG AA
ACTA2	Forward	GTG TTG CCC CTG AAG AGC AT
	Reverse	GCT GGG ACA TTG AAA GTC TCA
GAPDH	Forward	TCG TCA TCA ATG GAA GCC CC
	Reverse	AAA TGA GCC CCA GCC TTC TC

*VEGF* vascular endothelial growth factor, *VEGFR* vascular endothelial growth factor receptor, *NRP* neuropilin, *PECAM* platelet endothelial cell adhesion molecule, *Tie* tyrosine kinase with Ig-like loops and epidermal growth factor homology domains, *FN* fibronectin, *ACTA2* α-smooth muscle actin, *GAPDH* glyceraldehyde 3-phosphate dehydrogenase.

### Immunofluorescence staining of human TM tissues

All procedures involving human TM tissues were approved by the Kumamoto University Certified Clinical Research Review Board and were carried out in accordance with the Declaration of Helsinki. Written informed consent was obtained from all adult participants who had undergone ab interno trabeculotomy. TM tissues were obtained during trabeculotomy ab interno using a Kahook dual blade (KDB; JFC Sales Plan, Tokyo, Japan). Excised TM tissue was immediately fixed in 10% formaldehyde and embedded in paraffin for immunofluorescence microscopy. Five-micrometer-thick paraffin sections were deparaffinized, rinsed with deionized H_2_O, and incubated with 5% exosome-free FBS in PBS for 60 min at room temperature. The tissue sections were incubated with primary antibodies against CD81 and CD63 at 4 °C overnight. After washing with PBS, the sections were incubated with Alexa Fluor 488- or Alexa Fluor 594-conjugated secondary antibodies (Invitrogen-Molecular Probes, Eugene, OR, USA) for 60 min at room temperature. The sections were washed and mounted using VECTASHIELD containing 4′,6-diamidino-2-phenylindole (DAPI) (Vector Laboratories, Burlingame, CA, USA). Images were visualized using a BZ-X710 fluorescence microscope (Keyence, Osaka, Japan).

### Transmission electron microscopy

Human TM tissues were fixed with 2.5% glutaraldehyde and 2% paraformaldehyde in 0.1 M phosphate buffer, cut in the fixative, and additionally fixed at 4 °C for more than 2 h. Following washing, the tissues were postfixed in 1% OsO_4_ at 4 °C for 1 h. The tissues were washed and stained with 1.5% uranyl acetate at 4 °C for 1 h. After dehydration in ethanol and propylene oxide and substitution in resin and propylene oxide, the tissues were embedded in epoxy resin for 3 h and then polymerized at 60 °C for more than 48 h. The tissues were trimmed and cut into 60- to 70-nm sections. The tissues were stained with 1.5% uranyl acetate for 10 min and lead citrate for 10 min. Images were acquired at 80 kV on a HITACHI 7700 transmission electron microscope (Hitachi, Tokyo, Japan).

## Supplementary Information

Supplementary Information.

## Data Availability

The datasets generated and/or analyzed in this study are available in the National Center for Biotechnology Information Gene Expression Omnibus repository with an unique persistent identifier (NCBI tracking system accession number: GSE175464). The hyperlink to the datasets is https://www.ncbi.nlm.nih.gov/geo/query/acc.cgi?acc=GSE175464.

## Abbreviations

TM: Trabecular meshwork
SC: Schlemm’s canal
microRNA: MiRNA
exomiR: Exosomal microRNA
AH: Aqueous humor
EV: Extracellular vesicle
