# Supplementary material for: The effects of exosomes derived from trabecular meshwork cells on Schlemm’s canal endothelial cells
Source: Sci Rep. 2021 Nov 9;11:21942. doi: 10.1038/s41598-021-01450-9 (PMC8578291; doi:10.1038/s41598-021-01450-9)

Title page

Title: The effects of exosomes derived from trabecular meshwork cells on Schlemm's canal endothelial cells

Authors' names: Eri Takahashi*[1], Junji Saruwatari[2], Tomokazu Fujimoto[1], Yuki Tanoue[3], Takaichi Fukuda[4], Toshihiro Inoue[1]

1 Department of Ophthalmology, Faculty of Life Sciences, Kumamoto University, Kumamoto, Japan; 2 Department of Pharmacology and Therapeutics, Graduate School of Pharmaceutical Sciences, Kumamoto University; 3 International Research Center for Medical Sciences, Faculty of Life Sciences, Kumamoto University, Kumamoto, Japan; 4 Department of Anatomy and Neurobiology, Faculty of Life Sciences, Kumamoto University.

Corresponding author: Eri Takahashi, Department of Ophthalmology, Faculty of Life Sciences, Kumamoto University, 1-1-1 Honjo, Chuo-ku, Kumamoto 860-8556, Japan.

Telephone: +81 96 373 5247; Fax: +81 96 373 5249.

E-mail address: eritakahashi@kumamoto-u.ac.jp

Supplementary Figure 1: Full-length blots for Figure 1B

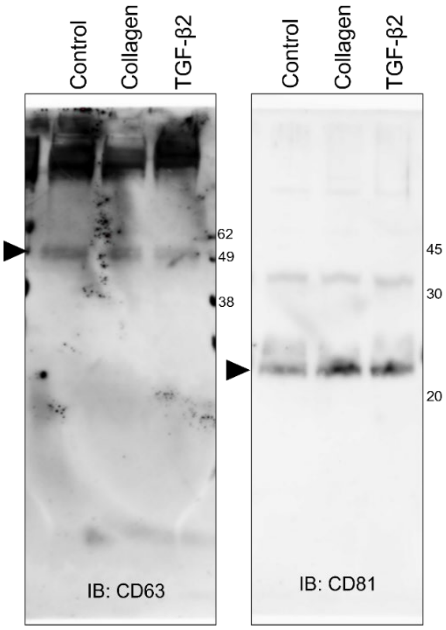

Supplementary Figure 2

A constructed network of 46 miRNAs and 1440 mRNAs in TGF-β2-stimulated cultures.

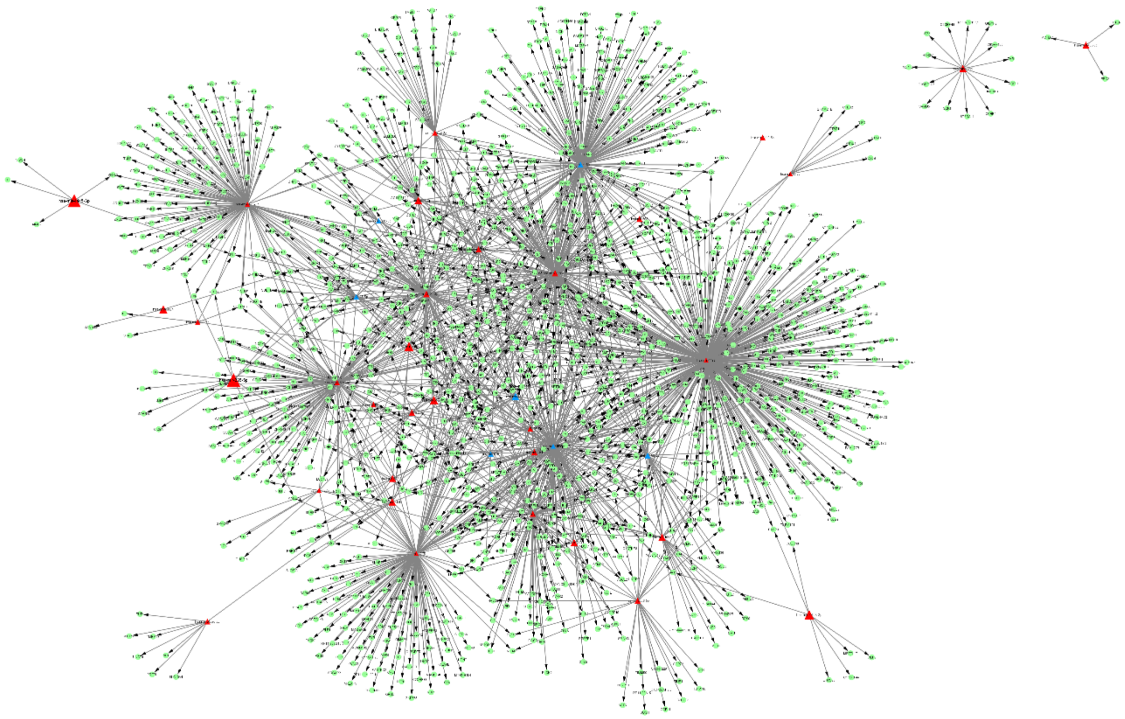

Supplementary Table S1: Genes associated with enriched GO and KEGG terms in the TGF-β2-treated group compared to the control group.

Category	ID	Term	-LOG10(P-value)	Genes
GOTERM	GO:0034097	Response to cytokine	5.91	JUND, AVPR2, PDX1, OSMR, DDOST, PML, RELB, GNAO1, MAPKAPK3, TIMP2, PGGT1B, RARA, TIMP1, JUNB, IL6R, BCL2L1
GOTERM	GO:0045944	Positive regulation of transcription from RNA polymerase II promoter	3.69	BACH1, ELK1, SOX2, CREB3L1, ZMIZ2, NFATC2IP, JUNB, CDK5RAP3, ACVR1, EOMES, LMO1, CASK, BHLHA15, EBF4, SFRP2, TBL1XR1, NCL, NOTCH1, GATA6, NHLH2, RAI1, ZNF821, RIPK1, ZBTB7C, SMAD4, JUND, BCL11B, SMAD7, LHX3, CCPG1, PIN1, SP7, OGT, ACVRL1, BARHL1, RARG, BMPR2, PTPRN, NR2E1, CYR61, IKBKB, MED12, SIX3, SUMO2, FEZF2, NCK1, MYOG, KDM6B, BCAS3, SS18, RBM14, PARP1, MKL1, PPP2R5B, EN2, SENP2, PLSCR1, RBL1, IFNG, MED21, IL1B, RARA, HDAC4, FOXC2, KDM3A, CEBPB, NUFIP1, SATB2, NR1I3, TAF1L, NR2C2, ACVR1B, ASCL1, RELB, MAPK7, TBX1, CDKN2B, NFYC, STAT3, PDX1, NR1H3, LILRB1, GTF2H1, MAPK14, USF1, PML, GRIN1, ASXL1, MEIS1, TFAP4, NFIA, TNIP2, WT1, TTC5, SSBP4, TAF1, NEUROG3
GOTERM	GO:0000122	Negative regulation of transcription from RNA polymerase II promoter	2.96	ZNF496, RARG, ZNF296, EPO, SUV39H1, NR2E1, NOC2L, BACH1, SOX2, MECP2, HMG20A, ANKRD2, FEZF2, JUNB, ZNF565, EOMES, LMO1, KLF11, HES6, HBZ, PARP1, DNMT3A, MAGEA2B, RUNX3, FOXP2, EID1, RBL1, BRMS1, IFNG, TBL1XR1, RARA, TXNIP, AJUBA, HDAC4, FOXC2, DNMT1, NOTCH1, SATB2, ARX, GATA6, GPS2, ASCL1, ASCL2, RELB, DNAJB5, ZBTB7A, RBM10, MBD3, HEXIM1, PLK3, CBX6, SMAD4, JUND, MBD1, SNAI3, STAT3, PDX1, NR1H3, ELP2, SMAD7, ASXL1, EFNA1, NFIA, WT1, TRPV4, BHLHE40, CALR, PKIG, RCOR3, ZNF136, FGFR1, NEUROG3
GOTERM	GO:0003682	Chromatin binding	2.93	HDAC4, CEBPB, DNMT1, SATB2, FMR1, SUV39H1, HP1BP3, ARX, GATA6, NOC2L, ASCL1, ELK1, RELB, MECP2, MED12, CREB3L1, ANKRD2, FEZF2, EOMES, KDM6B, MBD3, BCAS3, SMAD4, MBD6, NPM2, NCOA5, MBD1, CBX1, ACTL6A, DNMT3A, PDX1, GTF2H1, MLH1, REC8, MEIS1, POLR3A, NFIA, TTC5, CSNK2B, GMNC, TOP1, AJUBA, RAN, ASF1A
GOTERM	GO:0005515	Protein binding	2.67	IPO13, RNH1, RPL3, FRMPD4, PWWP2B, C2ORF88, ENO1, NOC2L, BACH1, ELK1, ZC3H12D, LIPE, COMTD1, NUB1, WDR92, PSMD2, ZMIZ2, CREB3L1, PRKACA, PRSS3, JUNB, ZNF169, CDK5RAP3, STARD3, RFX1, ATG13, GTPBP4, SND1, EML2, UNC13D, SIGIRR, NISCH, EID1, FAM133A, ACE2, EWSR1, RUFY4, CLDN8, CLDN7, TXNIP, RBCK1, ASF1A, PRR13, RHNO1, NLRX1, NAIF1, GATA6, PDGFA, NCAPG, AKAP17A, MAGEA9B, NYAP2, SULT1A1, HPX, CYB5R1, NCSTN, PPP4C, PRKAR2B, HOXC9, ZBTB7B, ZBTB7A, HEXIM1, GSTM4, SMCP, PLK3, NGFR, PRELID1, AFAP1L1, JUND, SIVA1, SETD1B, THAP11, ABTB1, ZNF136, FARSB, RNF220, ZNF496, PHF2, RARG, WDR48, ELL, MTMR14, RPLP0, LRRC4, CLTA, SNX11, FAM127B, WDR43, ARHGAP4, PQBP1, MECP2, IKBKB, MED12, TRMT112, FAM102A, C1QTNF8, TMSB4X, TMSB4Y, ENSA, RSPO2, TIMP2, GAST, TIMP1, LRRC47, MYOG, IKBIP, SS18, DUSP2, ACTL6A, ANXA5, MED27, CIT, PLSCR1, PSMA1, MED21, RARA, VAMP1, KIFC3, TKT, VAMP5, ARF5, CTDP1, SYNPO2, GCNT1, GPS2, ASCL1, PTGS2, GGA1, LMAN1, TCOF1, ALOX5, ADAM23, CLPTM1, FLNA, BRD9, STX4, ZNF343, MBD3, CDKN2B, IL10RB, MBD1, STAT3, CARD9, NR1H3, GTF2H1, FAM104A, RNF146, TTLL6, WT1, SNX17, TAOK1, SHKBP1, MORN3, PLP1, CCDC6, RIMBP3, LAMTOR1, LAMTOR3, EPO, FMR1, NAB2, HP, SAT2, TSEN2, RAB43, PPP1CB, MYBPHL, PIM1, MACROD1, ZNF207, CEP250, PIM3, MAP3K9, PIM2, ZNF687, LZTS2, AP2M1, USP5, FBXO18, MAGI3, MUL1, DYRK1B, VRK1, POLRMT, FOXP2, EDAR, ACLY, RINT1, MAPKAPK3, SLC9A7, RAB37, NCL, MADD, SNRPE, SNRPF, LY6H, TSSK2, AJUBA, DSC2, PFKFB2, CUL9, GRIA1, DNMT1, NOTCH1, UQCRB, FAM114A1, TROAP, SPATA2, FAM109B, SPATA4, FBXO46, GTF2E1, KLC4, DDRGK1, C1ORF109, PGK1, G3BP2, TNFRSF14, NDP, ZKSCAN4, SRGAP2, SOCS7, CD300LG, ZNF300, TRAPPC2, ZNF540, ANKRD28, UVRAG, KIRREL, ZBTB10, BCDIN3D, FOXN3, LSM3, COPS3, NSFL1C, SON, SP2, SLC26A8, PIN1, RLN3, LHX6, TUBGCP3, CNIH2, LRCH4, ZNF774, OGT, MCU, BCL2L1, SNAP25, CNTNAP1, BMP10, HSP90AB1, NXT1, BMPR2, CDCA7L, PTEN, DUSP14, METTL13, AP1G2, SNRNP70, SUMO2, ITGAX, HMG20A, ATP7A, METTL17, NCK1, EIF5A, BCAS3, GPX3, RFC2, AVPR2, DNMT3A, RAD23A, SPTB, ARFGAP1, DDOST, HAUS1, DNAJC1, PKM, SLC25A17, XCL2, POLR1D, MCM4, BIRC5, ITGA7, TMBIM6, TOP1, FXN, GAPDH, CNGB1, HDAC4, PPP1R15B, CEBPB, NUFIP1, DGCR14, C1S, SRC, NOD1, MLLT3, NTN1, LMF2, FYB, HDAC6, RAB21, SELENBP1, HIRIP3, NCLN, RAB25, DNAJB5, PHKG2, CACNG1, BEND7, ACSS1, POLR2J, UBQLN2, KCNJ3, PTPN18, CIZ1, GPKOW, KCNIP3, TXNRD1, PML, ASXL1, TNRC6C, RAD52, NIN, BRE, DNAJA2, STUB1, CUL4B, IFITM1, CPNE7, SLC23A1, HHIP, ACTB, MAGEA9, SOX2, TCL1A, LGALS1, C7ORF31, ARL6IP5, HABP4, DAG1, FBXO4, DPF2, ARL6IP4, CPNE2, SPA17, MAGEA6, TNS3, SCN1B, TBC1D22B, FBXO9, ACVR1, PDHX, ARRDC1, ACTN1, IFRD2, TM4SF4, RUNX3, SRRM1, POR, KATNAL1, CDC37, CSNK2B, BTK, UPRT, TRIM16, MGARP, KCTD17, NBL1, OBSL1, LLGL1, SMIM3, PDE1B, STXBP1, SP140, CD1B, NHLH2, WBSCR27, NUAK2, APOH, TSPAN6, SCAP, ZNF821, RBM10, RAB11FIP4, NTSR1, PPP1R26, FIS1, TMEM86B, BCL11B, SYNRG, INTS3, RCC2, CIDEB, IGFL1, SRPK1, PARP11, AIMP2, STAR, TH, RPL27A, NMU, TRMT12, INTS5, CALR, INTS9, RCOR3, RPS23, ACVRL1, DIRAS3, IFNA4, PTPRS, ACY1, GDI1, FKBP15, PTPRN, SUV39H1, STAB2, IKBKAP, IDE, LIMD2, BABAM1, PCM1, PPP3CC, NMRK2, ENC1, CTSG, CD37, SPTAN1, HBZ, PARP1, NCOA5, CCDC116, RHOG, COMMD3, AMPD2, NME4, HBA1, RHOC, VAV2, RHOB, ANO1, KXD1, ALDOC, KIF2C, FATE1, MATN4, STK40, FOXC2, RAMP2, GAGE12E, TAGLN, GAGE12C, GAGE12D, SATB2, HAAO, GAGE12B, IFI6, XIAP, TMPRSS2, PRKX, HSD17B8, RAD54B, GNAI2, RELB, ARVCF, C10ORF95, KIAA0753, LMNA, PAK6, DISC1, PCNT, GAGE12I, UBL4A, NPDC1, NPM2, GCH1, CD70, NFYC, HUWE1, KLHL2, LILRB2, MLH1, LILRB4, PEX14, GRIN1, SSX2B, BOP1, TFAP4, SEMG2, TTC5, KRT16, TACC3, RAB9A, KIAA1958, NEUROG3, RTKN, UBE3C, CD82, PAGE1, COMT, COMP, CHCHD4, PAIP2B, ARHGDIA, IL6R, KRT6B, SH3GL1, KRT6A, NANOS1, LMO1, HIST1H2AK, CASK, CDKAL1, PCOLCE, TUFM, LGALS9B, BRMS1, TBL1XR1, TMEM139, COL4A3, SCG5, PPIH, UBE2V1, CALCA, CCDC148, PXN, CACNA1B, DAPP1, LPL, PDS5B, PLD1, TIMM50, RAI1, IRAK2, RHPN1, RIPK1, UTP20, WNT3, ASIC1, FAM83A, HSPA9, CBX6, SMAD4, NUP155, CBX1, KLHL22, COQ9, TJAP1, SMAD7, EFNA1, PYY, CCPG1, HOOK1, ALPP, FGF19, TRPV4, BHLHE40, UBA1, FBN1, FGFR1, ZSCAN1, RTP4, EDA, HP1BP3, ATP2A2, ATP2A1, DMKN, LMNB1, SPRED2, RBM4, SENP7, SIX3, MLST8, FLOT1, ABCD1, UTP15, KDM6B, KLF11, RBM14, NCBP2, MKL1, PPP2R5B, MAGEA2B, SSTR2, SENP2, TGFBR1, GIP, DNM2, SUPV3L1, FAM189B, TMIGD2, PSRC1, RBL1, DPPA4, FAM96B, NAA35, LUC7L3, ATG4B, PFDN4, B4GALT7, LTK, USP11, BRAT1, RGS16, RBM4B, ASAP2, NR2C2, ACVR1B, LTC4S, PARL, CLN6, AURKA, MAPK7, TNKS2, PRRG2, MKNK2, C19ORF73, FAM63A, STOM, RALY, PDLIM5, DNAI1, KDM4A, USP26, SGTB, WTAP, CMTM5, KRTAP10-9, MX1, ZNF180, PDE4DIP, MAPK14, USF1, MEIS1, CD6, TNIP2, POLE3, SSBP4, CCDC172, RAN, TAF1
GOTERM	GO:0016020	Membrane	2.63	RERGL, IFITM1, FMR1, ZAN, SLC23A1, TNC, ICAM2, SLC4A2, ENO1, COMT, ACTB, ABHD13, STS, PSMD2, CREB3L1, PAPOLG, NIPAL2, ARL6IP5, DAG1, SPA17, PHLDA2, PRKACA, IL6R, CDK5RAP3, KRT6A, SLC12A9, ACVR1, MAGI3, BROX, MUL1, CDKAL1, GTPBP4, UNC13D, SND1, TUFM, TPST2, SIGIRR, NISCH, ACLY, CACNB3, POR, NCL, TSNARE1, PDGFA, ITPR2, NCAPG, PLA2G6, PLD1, GIGYF2, FUT4, FUT3, CYB5R1, NCSTN, PGK1, RCE1, ST3GAL1, CTSA, FIS1, OSBPL8, NOP14, TMEM86B, NUP155, RCC2, SUCO, SLC4A11, EFNA1, AIMP2, RPL27A, SLC26A8, INTS5, CNTN1, TUBGCP3, CALR, CLPTM1L, DHCR7, FARSB, RPS23, BCL2L1, CD320, ACVRL1, SNAP25, EDA, HSP90AB1, FKBP15, RPLP0, STON1, LMOD1, CLTA, ATP2A2, ATP2A1, DBH, MAGT1, LSP1, SLC2A6, C8A, RERG, LMNB1, PANX1, MED12, SPRED2, PCM1, AP1G2, ITGAX, FLOT1, RAC2, SDF4, CD37, ATP7A, SPTAN1, ABCD1, SCYL1, IKBIP, EIF5A, SERPINB1, PARP1, PCDHGA3, ELMOD2, ANXA5, EN2, HBA1, DDOST, TGFBR1, CIT, DNAJC1, PLSCR1, SLC25A17, IL3RA, CDHR3, MCM4, TMBIM6, KIF2C, ERGIC3, GAPDH, IL9R, GRAMD1B, KDM3A, LTK, CANT1, SLC20A1, PALM2, BRAT1, GCNT1, PTAFR, GDPD1, RFNG, ACVR1B, ATP2C1, LMF2, CLN6, GNAI2, SELENBP1, LMAN1, GGA1, NT5E, NCLN, MTHFD1L, CLPTM1, FLNA, STOM, NCAM1, STX4, PDLIM5, ATP6V0E2, FILIP1L, PCNT, IL12RB2, UBL4A, ATAD1, LMLN, PCDHGC3, HUWE1, LILRB2, PLEKHA4, MLH1, PEX14, POLR3A, CD6, SNX17, LPCAT4, DNAJA2, SLC28A2, FOLR1, RAN, PNPLA2
GOTERM	GO:0032924	Activin receptor signaling pathway	2.47	ACVR1, ACVRL1, BMP10, BMPR2, ACVR1B, TGFBR1
GOTERM	GO:0042127	Regulation of cell proliferation	2.41	NGFR, DNMT1, JUND, BMPR2, SRC, PTGER2, HP1BP3, ARX, TNC, XIAP, PDS5B, DBH, CXCL13, GRPR, TNFRSF10D, ACE2, SIX3, BTK, TXNIP, TNFRSF14, DHCR7, PLCD3, JUNB, RELT
GOTERM	GO:0007626	Locomotory behavior	2.36	B4GALT2, SNAP25, MCOLN3, PDE1B, PTEN, CACNA1B, HTR2C, DBH, MECP2, GNAO1, MEIS1, TH, FEZF2, ATP7A
GOTERM	GO:0043066	Negative regulation of apoptotic process	2.31	LTK, RARG, FOXE3, SRC, GATA6, PTEN, XIAP, NR2E1, ASCL1, CYR61, AURKA, SLC25A27, COMP, IKBKB, MAPK7, NUAK2, MPZ, ARHGDIA, PIM1, FLNA, PIM3, PIM2, TIMP1, NTSR1, HSPA9, NGFR, PLK3, PRELID1, BCL11B, ANGPT1, ANXA5, STAT3, PAFAH2, TNFRSF10D, HIGD2A, SUPV3L1, SON, LHX3, WT1, CLDN7, RARA, BIRC5, NAA35, TMBIM6, FXN, STK40, BCL2L1
GOTERM	GO:0030336	Negative regulation of cell migration	2.30	ACVRL1, OSBPL8, BMP10, IFITM1, PTEN, GTPBP4, RHOB, ARHGAP4, SMAD7, NISCH, SFRP2, KRT16, ARHGDIA, DAG1, EPPK1
GOTERM	GO:0005667	Transcription factor complex	2.18	MYOG, HES6, RBM14, SMAD4, RARG, FOXE3, JUND, PARP1, SNAI3, SATB2, GATA6, MED27, USF1, SMAD7, SOX2, NHLH2, MEIS1, RBL1, LHX3, ELMSAN1, RCOR3, AJUBA, JUNB, TAF1
GOTERM	GO:0010447	Response to acidic pH	2.16	SRC, ASIC3, GIP, ASIC1, LGMN
GOTERM	GO:0048286	Lung alveolus development	2.14	BMPR2, PKDCC, PDPN, PDGFA, ATP7A, TNS3, FOXP2, STK40
GOTERM	GO:0035264	Multicellular organism growth	2.11	RARG, PKDCC, WDR48, GIGYF2, ADD1, SP2, TNKS2, TBL1XR1, RARA, SLITRK6, DHCR7, UNC79, STK40
GOTERM	GO:0006915	Apoptotic process	2.09	RTKN, EPO, PTEN, NOC2L, COMP, LGALS1, WDR92, PIM1, FAM3B, DPF2, PIM3, PIM2, MAP3K9, PHLDA2, EIF5A, DFFB, KLF11, UNC5A, MAGI3, MUL1, TGFBR1, RHOB, MFSD10, EDAR, NISCH, PLSCR1, SFRP2, BRMS1, IFNG, IL1B, BIRC5, PDE1B, BRAT1, NAIF1, XIAP, NOD1, NTN1, C3ORF38, NUAK2, PAK6, RIPK1, RNF130, PLK3, PRELID1, KCNIP3, MX1, CIDEA, CIDEB, MAPK14, PML, AIMP2, BRE, TNIP2, CLPTM1L, OGT
GOTERM	GO:0007613	Memory	2.05	B4GALT2, ATAD1, CEBPB, TH, PTEN, DBH, PLA2G6, PTGS2, KCNK2, GIP, ASIC1
GOTERM	GO:0044389	Ubiquitin-like protein ligase binding	2.05	CEBPB, DDRGK1, PTPRN, CDK5RAP3
GOTERM	GO:0043195	Terminal bouton	2.05	CALCA, OPHN1, TH, SV2A, STXBP1, NMU, VAMP1, AP2M1, NTSR1, GRIN1, CNGB1
GOTERM	GO:0060740	Prostate gland epithelium morphogenesis	2.04	RARG, NOTCH1, TNC, CYP7B1
GOTERM	GO:0021902	Commitment of neuronal cell to specific neuron type in forebrain	2.04	BCL11B, SATB2, FEZF2, ASCL1
GOTERM	GO:0032609	Interferon-gamma production	2.04	EOMES, AVPR2, LILRB1, IL12RB2
GOTERM	GO:0012501	Programmed cell death	2.04	GSDMC, PKM, TOP1, RNF130
GOTERM	GO:0005581	Collagen trimer	2.02	EDA, PCOLCE, C1QL3, COL19A1, WDR33, FAM122B, COL2A1, C1QTNF8, C1QTNF7, COL4A3, COL6A6, TIMP1, EMID1, C1QC
GOTERM	GO:0051056	Regulation of small GTPase mediated signal transduction	2.01	GDI1, ARHGEF17, RHOG, RHOC, ARHGAP36, ARHGAP25, FGD1, RHOB, ARHGAP4, VAV2, ARHGAP31, ARHGEF9, DEPDC7, OPHN1, ARHGAP40, ARHGDIA, RAC2, SRGAP2
KEGG_PATHWAY	hsa04060	Cytokine-cytokine receptor interaction	2.43	IFNA4, BMPR2, EDA, IFNA6, EPO, CXCR6, CXCL13, ACVR1B, CCL1, TNFRSF14, RELT, IL6R, CCL16, IL12RB2, CCL15, ACVR1, NGFR, CCL24, IL10RB, CD70, OSMR, TGFBR1, TNFRSF10D, EDAR, IFNG, IL1B, IL3RA, XCL2, IL9R
KEGG_PATHWAY	hsa04750	Inflammatory mediator regulation of TRP channels	1.84	SRC, PTGER2, TRPV3, ITPR2, HTR2C, PLA2G6, MAPK14, PPP1CB, ASIC4, IL1B, TRPV4, PRKACA, ASIC3, ASIC1
KEGG_PATHWAY	hsa04921	Oxytocin signaling pathway	1.61	SRC, ITPR2, CACNA1D, PTGS2, ELK1, ACTB, GNAI2, GNAO1, PPP1CB, CACNB3, CACNG8, PPP3CC, MAPK7, CACNG1, PRKACA, MYL9, CACNG4, KCNJ3
KEGG_PATHWAY	hsa05020	Prion diseases	1.60	NOTCH1, IL1B, NCAM1, PRKACA, ELK1, C8A, C1QC
KEGG_PATHWAY	hsa04728	Dopaminergic synapse	1.59	GRIA1, CACNA1B, PPP2R5B, ITPR2, CACNA1D, COMT, MAPK14, GNAI2, GNAO1, PPP1CB, GNAL, PPP3CC, TH, CREB3L1, PRKACA, KCNJ3
KEGG_PATHWAY	hsa04010	MAPK signaling pathway	1.45	CACNA1B, PDGFA, CACNA1D, ELK1, RELB, IKBKB, CACNG8, PPP3CC, MAPK7, MKNK2, RAC2, FLNA, CACNG1, PRKACA, CACNG4, DUSP2, JUND, MAPK14, TGFBR1, CACNB3, MAPKAPK3, TAOK1, IL1B, FGF19, LAMTOR3, FGFR1
KEGG_PATHWAY	hsa05412	Arrhythmogenic right ventricular cardiomyopathy (ARVC)	1.45	CACNB3, CACNG8, LMNA, DAG1, ITGA7, CACNA1D, CACNG1, DSC2, CACNG4, ACTB
KEGG_PATHWAY	hsa01230	Biosynthesis of amino acids	1.28	PKM, ACY1, IDH2, PGK1, PGAM4, ALDOC, GPT, ENO1, TKT, GAPDH
KEGG_PATHWAY	hsa04623	Cytosolic DNA-sensing pathway	1.18	IKBKB, IFNA4, IFNA6, POLR3A, IL1B, POLR1D, RIPK1, POLR2H, MB21D1
KEGG_PATHWAY	hsa05031	Amphetamine addiction	1.12	GRIA1, PPP1CB, ARC, PPP3CC, , CREB3L1, CACNA1D, PRKACA, GRIN1
KEGG_PATHWAY	hsa00010	Glycolysis / Gluconeogenesis	1.09	ALDH3A2, PKM, ALDH1B1, PGK1, PGAM4, ALDOC, ENO1, ACSS1, GAPDH
KEGG_PATHWAY	hsa05152	Tuberculosis	1.04	HSPA9, PLK3, IFNA4, CEBPB, IFNA6, IL10RB, SRC, NFYC, CARD9, LSP1, MAPK14, FCGR3B, PPP3CC, IFNG, IRAK2, IL1B, ITGAX, HLA-DQA2

Supplementary Figure 3: Full-length blots for Figure 5B

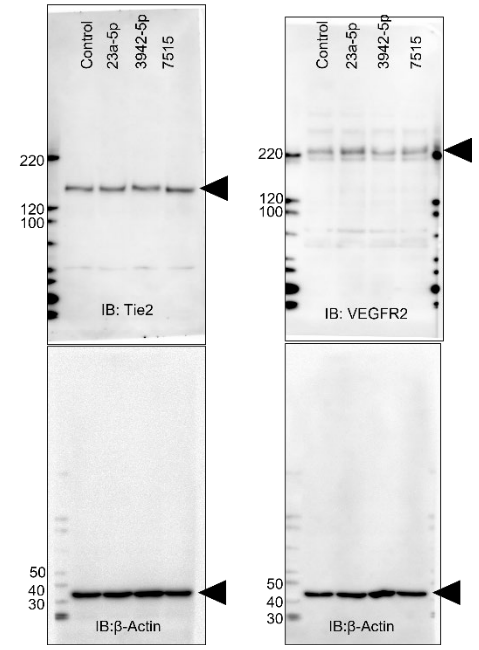

Supplement: Supplementary file 1 — Supplementary Information. [file 41598_2021_1450_MOESM1_ESM.pdf]
